# Molecular analysis of internal transcribed spacer 2 of *Dicrocoelium dendriticum* isolated from cattle, sheep, and goat in Iran

**DOI:** 10.1186/s12917-022-03386-2

**Published:** 2022-07-21

**Authors:** Ehsan Javanmard, Hanieh Mohammad Rahimi, Sara Nemati, Sara Soleimani Jevinani, Hamed Mirjalali

**Affiliations:** 1 Dept. of Medical Parasitology and Mycology, School of Public Health, Tehran University of Medical Sciences, Tehran, Iran; 2 Foodborne and Waterborne Diseases Research Center, Research Institute for Gastroenterology and Liver Diseases, Shahid Beheshti University of Medical Sciences, Tehran, Iran

**Keywords:** *Dicrocoelium dendriticum*, Livestock, ITS, Molecular analysis, Network analysis

## Abstract

**Background:**

*Dicrocoelium dendriticum* is a broadly distributed zoonotic helminth, which is mainly reported from domesticated and wild ruminants. There is little data covering the molecular features of this trematode; therefore, current study aimed to molecularly analyze *D. dendriticum* in livestock.

**Methods:**

Totally, 23 samples of *D. dendriticum* were collected from cattle, sheep, and goat from Ilam, Lorestan, and Khuzestan, three west and south-west provinces of Iran from February to August 2018. After genomic DNA extraction, the internal transcribed spacer (ITS) 2 fragment was amplified and sequenced in samples. To investigate genetic variations through the ITS 2 fragment of obtained *D. dendriticum,* phylogenetic tree and network analysis were employed.

**Results:**

All 23 samples were successfully amplified and sequenced. Phylogenetic tree showed that our samples were clearly grouped in a clade together with reference sequences. There was no grouping based on either geographical regions or hosts. Network analysis confirmed the phylogenetic findings and showed the presence of nine distinct haplotypes, while our samples together most of sequences, which were previously submitted to the GenBank, were grouped in the Hap1.

**Conclusions:**

Our findings indicated that although ITS 2 fragment discriminate *D*. *dendriticum*, this fragment is not suitable to study intra-species genetic variations. Therefore, exploring and describing new genetic markers could be more appropriate to provide new data about the genetic distribution of this trematode.

## Background

*Dicrocoelium dendriticum* is a broadly distributed zoonotic helminth in many areas, particularly those regions with animal husbandry [1, 2]. *Dicrocoelium dendriticum,* which is known as the small liver fluke or lancet liver fluke, is an imperative species in medicine, veterinary sciences, and economic industries [3, 4]. Various range of definitive hosts, mainly domesticated and wild ruminants, have been reported to harbor this trematode. *Dicrocoelium dendriticum* has a complex life cycle consist of three hosts including ruminants as definitive hosts and two invertebrate intermediate hosts (terrestrial snails as the first and formicid ants as the second intermediate hosts) [5]. Although infection by *D. dendriticum* is frequently observed in domesticated ruminants, reports of dicrocoeliasis in humans are rare, and this disease is classified as a neglected parasitic disease (NPD) [6, 7]. This disease can cause diarrhea, flatulence, biliary obstruction, cholangitis, acute urticaria and a serious liver problem, cirrhosis [8, 9]. Nevertheless, reports of spurious dicrocoeliasis are associated with consumption of undercooked infected liver of animals [10].

Dicrocoeliosis, as a foodborne zoonotic disease, caused by three species of *Dicrocoelium*, namely *D. dendriticum*, *D. hospes,* and *D. chinensis*, which involve the bile ducts and gall bladder of their hosts [12]. *Dicrocoelium dendriticum* has been reported in Europe, Asia, northern Africa, and North America, *D. hospes* is endemic in sub-Sahara and West Africa, and *D. chinensis* in Eastern Asia and Europe [3].

Although there are studies reporting *D. dendriticum* from ruminants [15–18], there is little data about the molecular analysis of this trematode in Iran [17, 19]. The prevalence of *D. dendriticum* was reported 5.68% and 2.13% in cattle and sheep, respectively, in an abattoir from Sabzevar, northeast of Iran [18]. In addition, 0.1% of condemn cattle livers from slaughterhouses in Sistan-Baluchestan province, southeast of Iran, were infected by *D. dendriticum* [20]. This platyhelminth is highly distributed in coastal provinces of the Caspian Sea [5]. *Dicrocoelium dendriticum* was reported from 36.72% of sheep and 6.09% of cattle in Guilan province, and 22.4% of sheep and 3.91% of cattle in Mazandaran province [5]. This study aimed to molecularly analyze *D. dendriticum* in three livestock ruminants including cattle, sheep, and goats based on amplification and sequencing of the internal transcribed spacer (ITS) 2 fragment of the ribosomal RNA (rRNA) gene in west regions of Iran.

## Methods

### Ethics approval and consent to participate

Verbal consent was taken from animal’s owners. Samples were taken from slaughtered animals for meat production in an abattoir in study regions. All experimental protocols were approved by the Research Institute for Gastroenterology and Liver Diseases and all procedures performed in this study were approved by the ethical standards (IR.SBMU.RIGLD.REC.1396.164) released by Ethical Review Committee of the Research Institute for Gastroenterology and Liver Diseases, Shahid Beheshti University of Medical Sciences, Tehran, Iran. In addition, all methods were carried out in accordance with relevant guidelines and regulations, and all authors complied with the ARRIVE guidelines.

### Sample collection

**Fig. 1:**
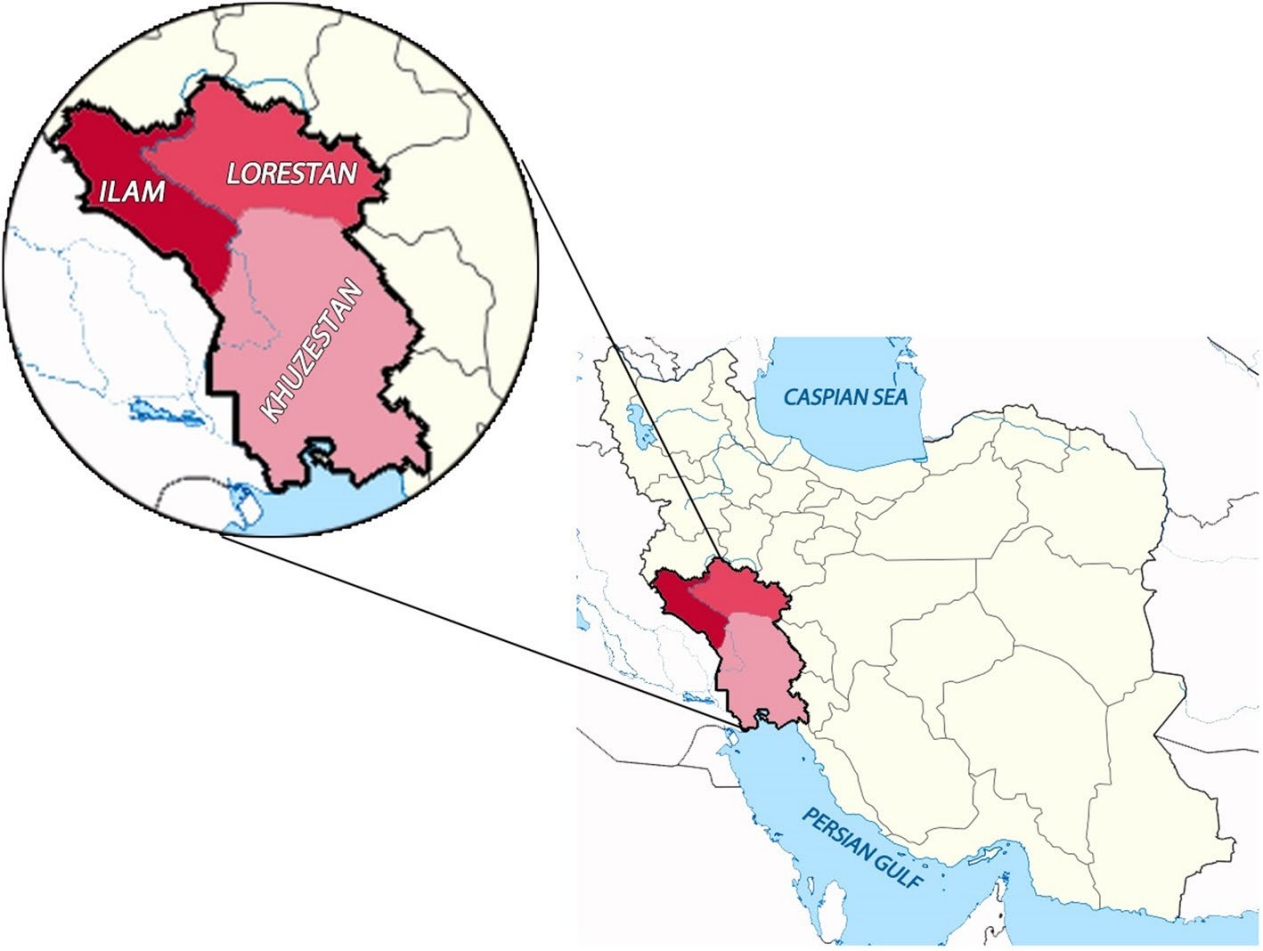
The map of Iran and sample collection sites

Totally, 23 samples of *D. dendriticum* were collected from the liver and gall bladder of sheep, cattle, and goats in some parts of Ilam, Lorestan, and Khuzestan provinces, west and south-west of Iran, from February to August 2018 (Fig. 1). Accordingly, samples were obtained from five, four, and three sheep, cattle, and goat, respectively. Except one of sheep, all other hosts provided two helminthes. Adult worms were carefully washed in PBS buffer (pH 7.4) and stored in -20ºC until use. All isolated worms were morphologically investigated based on their body length and orientation of testes [21].

### DNA extraction, PCR, and sequencing

DNA extraction was carried out for each worm using tissue DNA extraction kit (YektaTajhiz Azma [YTA], Tehran, Iran) according the manufacturer’s recommendations. Afterward, the discriminative fragment of the ITS 2 gene was amplified using the primers described as ITS2F: 5′-TGTGTCGATGAAGAGCGCAG-3′ and ITS2R: 5′-TGGTTAGTTTCTTTTCCTCCGC-3′ to amplify a ~ 480-bp fragment []. The amplification reactions were carried out in a 30 µl volume containing 15 µL of 2X Taq Red matermix (1.5 mM MgCl_2_; Ampliqon, Denmark), 10 ρM of each primer, and 2µL of template DNA. Distillated water was considered as negative control. PCR reactions were performed under the following conditions: one 94ºC cycle for 5 min, followed by 94 ºC for 45 s(denaturation), 56 ºC for 45 s (annealing), and 72 ºC for 45 s (extension). All three steps were repeated for 35 cycles with a final extension at 72ºC for 5 min. All PCR products were electrophoresed on 1.2% agarose gel and visualized by ethidium bromide (0.5 mg/ml) under ultraviolet illumination. To characterize haplotypes, all positive PCR results were sent for sequencing (ABI 3130 sequencers). All generated sequences were submitted to the GenBank database with accession numbers: OL455743 to OL455765.

### Sequence analysis, haplotype networking, and phylogenetic analysis

All nucleotide sequences obtained in the present study were compared to the basic local alignment search tool (BLAST) search (http://www.ncbi.nlm.nih.gov/blast/), and then were aligned using ClustalW [23] incorporated in BioEdit v.7.2.6. The phylogenetic tree and network analysis were performed to investigate the similarities and to visualize the relationship among haplotypes from different geographical regions and hosts of our *D. dendriticum* samples together with previously deposited sequences in the GenBank database from Iran and other countries. Haplotype network was constructed using PopART software [24]. A TCS network [25], based on parsimony method, was generated for the aligned haplotype sequences. In order to provide visual information about relationships among all samples, the phylogenetic was constructed based on the Maximum-likelihood (ML) and Tamura 3-parameter using the MEGA 6.0 [26]*.* The reliabilities of the trees were assessed using the bootstrap analysis with 1,000 replications.

## Results

### Morphological characterization and phylogenetic tree

**Fig. 2:**
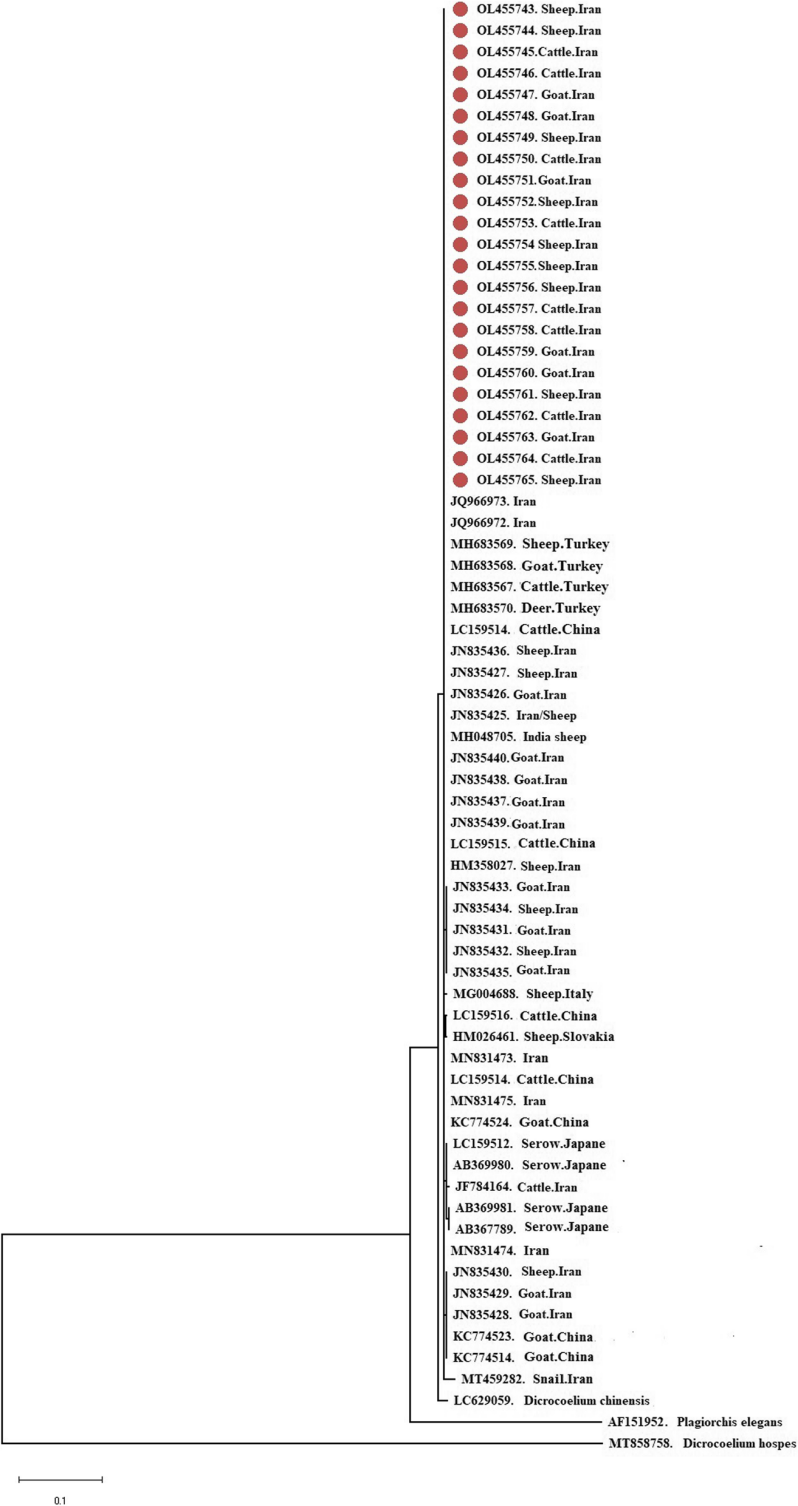
Phylogenetic tree of the ITS 2 fragments of rRNA gene for *D. dendriticum* isolated from livestock animals together with reference sequences. The phylogenetic tree represents that all sequences were grouped together and with the reference sequences. The phylogenetic tree was drawn using the maximum-likelihood method and the Tamura 3-parameter model. Bootstrap supports values of > 75% are indicated above the branches. Our sequences are indicated with maroon circle

All worms were morphologically investigated for their body length and orientation of testes, which all were *D. dendriticum.* The target fragment was successfully amplified and sequenced in all morphologically characterized samples. BLAST comparison showed that all sequences were *D. dendriticum*. The phylogenetic analysis showed that the samples obtained in the present study were clearly grouped in same clade with reference sequences. As seen in the tree, no grouping was found based on geographical origins and hosts (Fig. 2 A and B). All obtained sequences were similar in the sequenced fragment of the ITS 2 gene, but there were 18 polymorphism sites compared to those sequences of *D. dendriticum* previously submitted to the GenBank database (Table 1).

**Table 1:** Polymorphism sites and nucleotides in aligned sequences of the ITS 2 fragment from previously published studies compared to the sequences obtained in the current study

No	Accession numbers	Single nucleotide polymorphism sites																	
		**7**	**24**	**90**	**91**	**180**	**183**	**199**	**211**	**216**	**238**	**262**	**263**	**300**	**314**	**315**	**330**	**331**	**351**
**1**	**HM358027**	Deletion-A	Deletion-A	-	-	-	-	-	-	-	-	-	-	-	-	-	-	-	-
**2**	**JN835430**	-	-	-	-	T-C	A-T	A-G	-	-	-	-	-	-	-	-	-	-	-
**3**	**KC774523**	Deletion-A	Deletion-A	-	-	-	-	-	-	-	Deletion-A	-	-	-	-	-	-	-	-
**4**	**KC774524**	Deletion-A	Deletion-A	-	-	-	-	-	-	-	Deletion-A	-	-	-	-	-	-	-	-
**5**	**KC774514**	Deletion-A	Deletion-A	-	-	-	-	-	-	-	Deletion-A	-	-	-	-	-	-	-	-
**6**	**MN831473**	Deletion-A	Deletion-A	-	-	-	-	-	-	-	-	-	-	-	-	-	-	-	-
**7**	**MN831474**	Deletion-A	Deletion-A	-	-	-	-	-	-	-	-	-	-	-	-	-	-	-	-
**8**	**AB369980**	Deletion-A	Deletion-A	-	-	-	-	-	-	-	Deletion-A	-	-	-	-	-	-	-	-
**9**	**MT459282**	Deletion-A	Deletion-A	-	-	-	-	-	-	-	-	-	-	-	-	-	-	-	-
**10**	**JN835435**	-	-	T-C	Deletion-T	-	-	-	-	-	-	-	-	-	-	-	-	-	-
**11**	**JN835432**	-	-	T-C	Deletion-T	-	-	-	-	-	-	-	-	-	-	-	-	-	-
**12**	**JN835431**	-	-	T-C	Deletion-T	-	-	-	-	-	-	-	-	-	-	-	-	-	-
**13**	**JN835433**	-	-	T-C	Deletion-T	-	-	-	-	-	-	-	-	-	-	-	-	-	-
**14**	**JN835434**	-	-	T-C	Deletion-T	-	-	-	-	-	-	-	-	-	-	-	-	-	-
**15**	**JN835429**	-	-	-	-	-	-	A-G	-	-	-	-	-	-	-	-	-	-	-
**16**	**JN835428**	-	-	-	-	-	-	A-G	-	-	-	-	-	-	-	-	-	-	-
**17**	**MG004688**	-	-	-	-	-	-	-	A-T	-	-	-	-	-	-	-	-	-	-
**18**	**MT459282**	-	-	-	-	-	-	-	-	C-T	-	G-T	-	-	-	-	-	-	C-A
**19**	**LC159516**	-	-	-	-	-	-	-	-	-	-	-	A-T	-	-	-	-	-	-
**20**	**HM026461**	-	-	-	-	-	-	-	-	-	-	-	A-C	-	-	-	-	-	-
**21**	**AB369981**	-	-	-	-	-	-	-	-	-	-	-	-	A-G	-	-	-	-	-
**22**	**AB367789**	-	-	-	-	-	-	-	-	-	-	-	-	A-G	-	-	-	-	-
**23**	**LC159512**	-	-	-	-	-	-	-	-	-	-	-	-	-	G-A	-	-	-	-
**24**	**JF784164**	-	-	-	-	-	-	-	-	-	-	-	-	-	G-A	G-A	C-A	G-A	-

### Network analysis

**Fig. 3:**
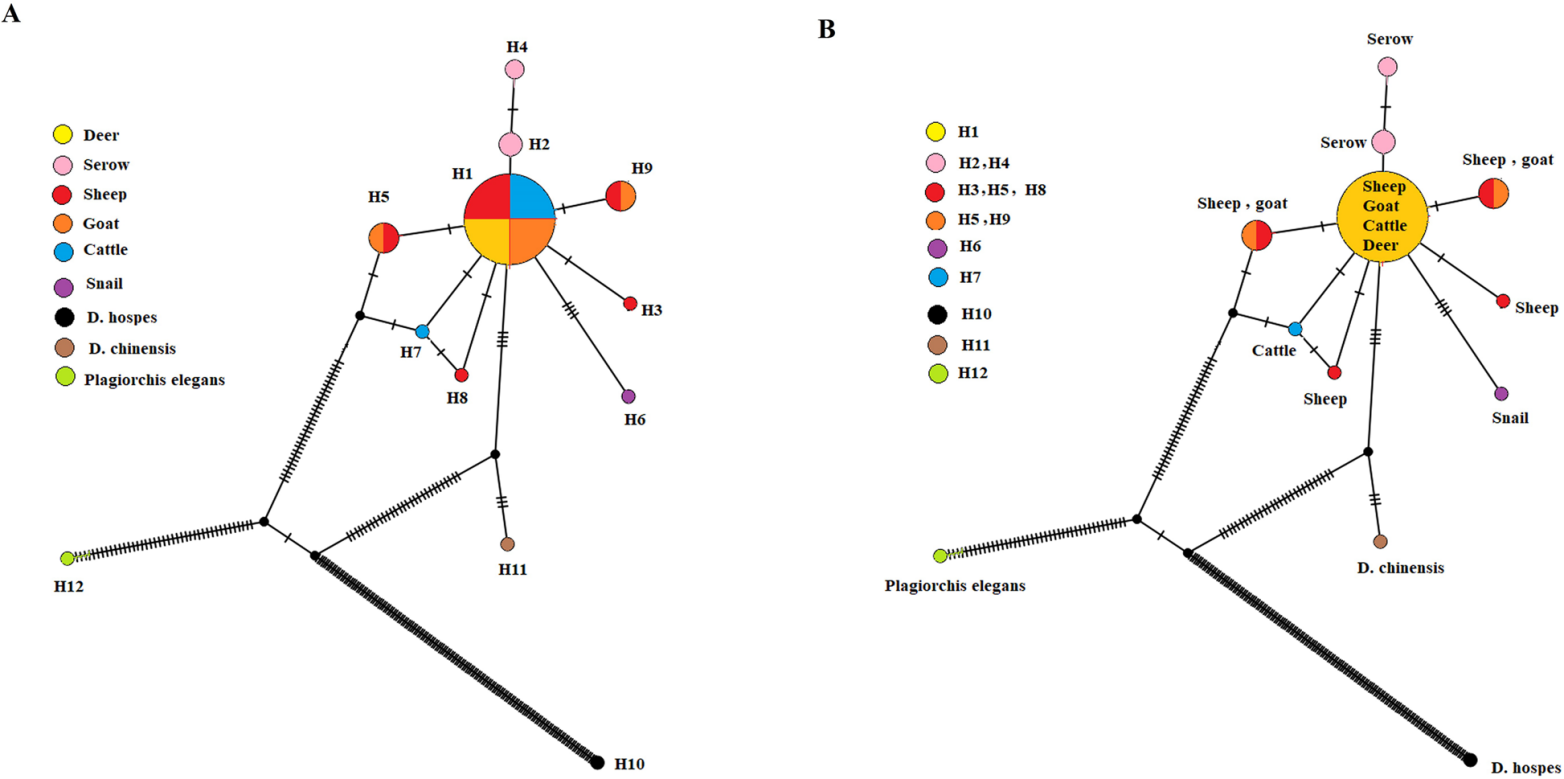
Network analysis shows clusters of haplotypes for each *D. dendriticum* sequences based on **A** hosts and **B** haplotypes. Most of sequences are grouped as Hap 1. Each color indicates certain hosts. *Plagiorchis elegans*, which is indicated with black circle, is outgroup

The result of network analysis confirmed the finding of the phylogenetic tree and showed the presence of nine distinct haplotypes among *D. dantriticum*. All of our samples together (Acc no. OL455743 to OL455765) with most of sequences from other studies were grouped in the Hap-1, which consisted of sequences from different geographical regions and hosts. Two isolates originated from Japan, which were isolated from Japannes serow, were grouped as two haplotypes; Hap-2, Hap-4. *D. dendriticum* isolated from sheep and goat in Iran, located in a distinct cluster; Hap-5, and snail isolate grouped as Hap-6 with significant variable sites (Fig. 3 and Table 2).

**Table 2:** The accession numbers and the number of dedicated sequences in each haplotype

Haplotypes	Accession numbers	Species
H1	(OL455743- JQ966973), MH683569, MH683568, MH683567, MH683570, LC159514, JN835436, JN835427, JN835426,. JN835425, KC774524, MN831473, MN831474, MH048705, JN835440, JN835438, JN835437, JN835439, LC159515, and MN831475	*D. dendriticum*
H2	LC159512, JF784164, and AB369980	*D. dendriticum*
H3	MG004688	*D. dendriticum*
H4	AB369981 and AB367789	*D. dendriticum*
H5	JN835430, JN835429, JN835428, KC774523, and KC774514	*D. dendriticum*
H6	MT459282	*D. dendriticum*
H7	LC159516	*D. dendriticum*
H8	HM026461	*D. dendriticum*
H9	JN835435, JN835432, JN835431, JN835433, and JN835434	*D. dendriticum*
H10	MT858758	*D. hospes*
H11	LC629059	*D. chinensis*
H12	AF151952	*Plagiorchis elegans*

## Discussion

*Dicrocoelium dendriticum* is a distributed platyhelminth, which has been mostly reported from ruminants, almost from all regions of Iran [16, 20, 27]. Historical evidence of *D. dendriticum* eggs in an ancient cemetery located in the Kiasar archeological site, north of Iran (247 BC-224 AD) [28], and a bronze age cemetery in west of Iran, from pre-Persepolis period [29], indicate the accompaniment of this fluke with humans from the beginning of animal domestication up to now.

Plenty of studies have reported *D. dendriticum* from different livestock in Iran based on parasitological techniques [17, 18, 27, 30, 31]. However, few molecular studies have analyzed *D. dendriticum* [6, 32]. Several molecular markers have been engaged to differentiate *Dicrocoelium* at species and strain levels [12, 17, 33]. Fragments of nuclear ribosomal genes and mitochondrial loci DNA have been evaluated using molecular techniques for Dicrocoeliid parasites [21, 33–36]. Molecular analysis of NADH dehydrogenase (NAD)1 gene suggested that this genetic marker was not suitable for molecular characterization of *D. dendriticum* [18]. On the other hand, although it was suggested that ribosomal cisteorn DNA and cytochrome oxidase subunit 1 (cox1) seem to be appropriate to explore intra-species variations through *D. dendriticum*, the number of reference sequences for these genes is not too much to interpret the probable correlation of genetic variations with hosts and origin of *Dicrocoelium* [12]; therefore, many studies have employed ITS fragments for genetic scrutinizing of this trematode.

Actually, rRNA gene is a suitable and widely employed marker for molecular analysis of nematodes, trematodes, and cestodes [37–39]. The ITS fragmnet of the rRNA gene and mitochondrial cox1 gene are used to distinguish trematodes [32]. Furthermore, the ITS 2 fragment of rRNA gene has been known as a reliable genetic marker used for molecular studies of flatworms [40–42]. In the current study, based on analysis of ITS 2 fragment, nine distinct haplotypes among *D. dendriticum* isolates comprised of our sequences together with previously deposited sequences from all the world were seen. In addition, phylogenetic analysis showed that all our sequences were grouped in one clade together with most of previously submitted sequences. In the line of our findings, in a study performed by Liu et al., ITS and mitochondrial genome of *D. dendriticum* and *D. chinensis* were amplified and sequenced, and showed that both fragments were discriminative enough to separate two species, but shared limited variations within each species [36]. Moreover, in a study carried out on sheep and goat in China, it was demonstrated that phylogenetic analysis of ITS 2 fragment was not able to separate *D. dendriticum* isolates based on hosts and origin [33]. A study in Iran was also failed to document significant intra-species variations in *D. dendriticum* based on molecular investigation of ITS fragment [19]. These results are supported by other studies indicating high similarity through rRNA gene of *D. dendriticum* isolated from all over the world [43]. Furthermore, it was reported that the although the divergence of ITS 2 fragment between *D. chinensis* and *D. dendriticum* was about 3.8%, the intra-species diversity was very low, ranging from 0–1.3% [21]. Therefore, genetic variations through the rRNA gene are suggested to be appropriate enough for inter-species analysis of *Dicrocoelium* spp., without considering geographical distribution and original hosts [14]. Indeed, the low intra-species differences regarding the hosts and geographical areas was previously reported that suggested genetic stability within the species of *Dicrocoelium* [21].

## Conclusion

Although ITS 2 fragment discriminate *Dicrocoelium* spp., at species level, our study represented a high similarity of ITS 2 fragment among *D. dendriticum* obtained from cattle, sheep, and goat from all sampling sites. Therefore, this fragment seems not to be suitable to study intra-species genetic variations. Taken together, in contrast to the well-known trematode, *Fasciola* spp., molecular data about *Dicrocoelium* spp., is limited and available genes such as rRNA, cox, and NAD1 seems not to be suitable for explore molecular variations in *Dicrocoelium* spp., regarding hosts and geographical area. However, characterization of different genetic markers can provide a clue about genetic distribution of this trematode.

## Data Availability

All generated data from the current study are included in the article.
